# PCR detection of *Burkholderia multivorans* in water and soil samples

**DOI:** 10.1186/s12866-016-0801-9

**Published:** 2016-08-12

**Authors:** Charlotte Peeters, Stijn Daenekindt, Peter Vandamme

**Affiliations:** 1 Laboratory of Microbiology, Department of Biochemistry and Microbiology, Ghent University, Ghent, Belgium; 2 Department of Sociology, Ghent University, Ghent, Belgium; 3 Department of Public Administration and Sociology, Erasmus University Rotterdam, Rotterdam, The Netherlands

**Keywords:** *Burkholderia multivorans*, PCR assay, *recA*, Environmental samples

## Abstract

**Background:**

Although semi-selective growth media have been developed for the isolation of *Burkholderia cepacia* complex bacteria from the environment, thus far *Burkholderia multivorans* has rarely been isolated from such samples. Because environmental *B. multivorans* isolates mainly originate from water samples, we hypothesized that water rather than soil is its most likely environmental niche. The aim of the present study was to assess the occurrence of *B. multivorans* in water samples from Flanders (Belgium) using a fast, culture-independent PCR assay.

**Results:**

A nested PCR approach was used to achieve high sensitivity, and specificity was confirmed by sequencing the resulting amplicons. *B. multivorans* was detected in 11 % of the water samples (*n* = 112) and 92 % of the soil samples (*n* = 25) tested. The percentage of false positives was higher for water samples compared to soil samples, showing that the presently available *B. multivorans recA* primers lack specificity when applied to the analysis of water samples.

**Conclusions:**

The results of the present study demonstrate that *B. multivorans* DNA is commonly present in soil samples and to a lesser extent in water samples in Flanders (Belgium).

**Electronic supplementary material:**

The online version of this article (doi:10.1186/s12866-016-0801-9) contains supplementary material, which is available to authorized users.

## Background

The *Burkholderia cepacia* complex (Bcc) represents a group of closely related [1, 2] and extremely versatile bacteria that can be applied for a range of bioremediation, plant growth promotion and biocontrol purposes [3]. Yet, these bacteria are simultaneously rare but important opportunistic pathogens in cystic fibrosis (CF) patients [4, 5]. The most prevalent Bcc CF pathogens are *Burkholderia cenocepacia* and *Burkholderia multivorans*. The low number of outbreaks caused by *B. multivorans* [6–8], and the fact that *B. multivorans* isolates from CF patients commonly represent unique strains, suggest that there is only limited person-to-person transmission, and that strains are acquired from non-human sources such as the natural environment [9]. Although many studies have described the isolation of *B. cenocepacia* from rhizosphere samples [10], only few reported on the occasional isolation of *B. multivorans* from environmental samples [11–14]. Consequently, the true environmental niche of *B. multivorans* is considered unknown. The fact that the few environmental *B. multivorans* isolates that have been reported mainly originated from water samples [12, 14] suggested that water is the most likely environmental niche of *B. multivorans*.

PCR-based diagnostic tests have been developed for Bcc species identification based on both the 16S rRNA and *recA* genes, resulting in two sets of species-specific primers for *B. multivorans* [15, 16]. Miller et al. [17] used the 16S rRNA based PCR assays for the culture-independent detection of Bcc in soil environments. In the latter study, none of the samples that were Bcc PCR positive yielded Bcc isolates, suggesting that cultivation-dependent methods for the detection of Bcc bacteria may underestimate environmental populations.

In the present study, we used a cultivation-independent *recA*-based PCR assay to assess the presence of *B. multivorans* in water and soil samples in Flanders, Belgium. Our results show that *B. multivorans* DNA could be detected only occasionally in water samples but to a greater extent in soil samples, and that the available *B. multivorans recA* primers lack specificity, especially when applied to the analysis of water samples.

## Results

### Specificity of the 16S rRNA and *recA* PCR assays

Preliminary experiments were performed to compare the specificity of the *B. multivorans*-specific primers that were available from previous studies [15, 16]. Therefore, PCR assays based on the *recA* and 16S rRNA genes were applied to DNA extracts of two water samples and the resulting amplicons were sequenced. The obtained sequences were analyzed using the NCBI blastn suite (blast.ncbi.nlm.gov) to evaluate specificity. For amplicons of the *recA* PCR assay, hits showed 98 % or more similarity with the *recA* sequence of *B. multivorans* ATCC 17616 (CP000868) (this degree of variability in *recA* gene sequences corresponds with the sequence diversity commonly observed within Bcc species [18]). For the 16S rRNA nested PCR, amplicons showed the highest similarity (96 %) with sequences of *Comamonadaceae sp.* (FM886892), *Ideonella sp.* (FM886860), *Roseatales sp.* (JQ917995), and *Mitsuaria sp.* (JQ659937), all belonging to the order *Burkholderiales*, yet demonstrating that the nested 16S rRNA PCR assay was not *B. multivorans* specific. Therefore, only the *B. multivorans* nested *recA* PCR assay was further optimized (i.e., PCR mix, BSA concentration, number of cycles, primer concentration and quantity of pooled PCR product being transferred to the second round of PCR; data not shown) and used to analyze all environmental samples.

### Detection of *B. multivorans* in environmental samples

All 112 water samples yielded PCR-grade DNA extracts without the need of an extra purification step. *B. multivorans* DNA was detected in 12 water samples (11 %), of which one sample (W132) already yielded a visible amplicon in the first round of PCR (showing 97 % similarity to the *recA* sequence of *B. multivorans* ATCC 17616). For the soil samples (*n* = 27), four yielded PCR-grade DNA without the need of an extra purification step, 21 samples needed extra purification using agarose plugs, and for two soil samples (S6 and S14) no PCR-grade DNA extract could be obtained. The latter soil samples were therefore excluded from the dataset. For soil DNA extracts containing 50 ng/μl or more DNA, an OD_320/260_ ratio of 0.15 or higher was a good indicator for the necessity of an extra purification step to obtain PCR-grade DNA. *B. multivorans* DNA was detected in 23 soil samples (92 %), of which none yielded a visible amplicon in the first round of PCR.

### Statistical data analysis

The first model tested if type (water, soil) and class (SRW, CRS, O) were significant predictors for *B. multivorans* detection. Binomial logistic regression showed that the type of sample (water versus soil) was indeed a significant predictor for *B. multivorans* detection (Table 1). Adding class to the model did not significantly improve the model (data not shown). The odds ratio from this model predicted that there was a 96 times (e^4.56^ = 95.58) bigger chance to detect *B. multivorans* in soil than in water samples.

**Table 1:** Binomial logistic regression model 1 (water and soil samples)

	Beta (SE)	Odds ratio (95 % CI)
Intercept	−2.12^***^ (0.31)	0.12 (0.06; 0.21)
Type		
Water (ref)	—	—
Soil	4.56^***^ (0.80)	95.83 (24.48; 646.70)
Chi^2^	65.50^***^ (df = 1)	

Binomial logistic regression with Bm as outcome and Type as predictor

Signif. codes: 0‘^***^’ 0.001‘^**^’ 0.01‘^*^’ 0.05 ‘.’

*SE* standard error, *CI* confidence interval

To test which characteristics of the water environment affected the presence of *B. multivorans*, a second model was tested in which pH, temperature and class were assessed as predictors for *B. multivorans* detection. Binomial logistic regression showed that pH and class were significant predictors for *B. multivorans* detection in water samples (Table 2). Model 2a includes the main effects of all the variables and shows that the probability of detecting *B. multivorans* was higher in water samples with high pH. The results also show that the probability of detecting *B. multivorans* was higher in streams (CRS) compared to swimming and recreational waters (SRW). Model 2b includes an additional interaction effect between temperature and pH to test whether the effect of pH on detection depends on the temperature. The model fit of model 2b improved and the interaction effect between temperature and pH indicated that the positive effect of pH was stronger when the temperature of the water was higher.

**Table 2:** Binomial logistic regression model 2 (water samples)

	Model 2a	Model 2b	
	Beta (SE)	Beta (SE)	Odds ratio (95 % CI)
Intercept	−2.72^***^ (0.59)	−3.51^***^ (0.82)	0.03 (0.00; 0.01)
pH	0.97 . (0.52)	1.70^*^ (0.69)	5.48 (1.52; 23.84)
Temperature	−0.22 (0.16)	−0.19 (0.16)	0.83 (0.59; 1.13)
Class			
SRW (ref)	—	—	—
O	−0.18 (0.92)	0.16 (0.96)	1.17 (0.14; 7.29)
CRS	1.47 . (0.80)	1.95^*^ (0.94)	7.06 (1.21; 52.19)
Interaction			
pH^*^Temperature	—	0.39^*^ (0.20)	1.47 (1.01; 2.25)
Chi^2^	8.24 . (df = 4)	12.36^*^ (df = 5)	

Binomial logistic regression with Bm as outcome and pH, Temperature and Class as predictor, without (model 2a) and with (model 2b) interaction effect between pH and Temperature

Signif. codes: 0‘^***^’ 0.001‘^**^’ 0.01‘^*^’ 0.05 ‘.’

*SE* standard error, *CI* confidence interval, *SRW* swimming or recreational water, *CRS* canal-river-stream, *O* other

### Detection limit *recA* PCR assay

The *recA* PCR assay was applied to serial dilutions of genomic DNA from *B. multivorans* R-20526 to determine its detection limit. The highest 50× dilution testing positive was the fourth one, from which a twofold dilution series was made. The highest twofold dilution testing positive in all three replicate runs of the assay was the second one, containing 1.35 genome equivalents/μl. Given that *recA* is a single-copy gene, and that for each dilution 2× 2 μl was added to the first round of PCR (set up in duplicate), the detection limit was determined to be 5.41 *recA* copies.

## Discussion

Because the goal of the present study was to gain insight in the environmental niche of *B. multivorans* in a time and cost-effective way, we chose for a conventional PCR method with gel-based detection of the resulting amplicons, instead of quantitative PCR. *B. multivorans*-specific primers based on both 16S rRNA and *recA* sequences were available from previous studies [15, 16]. Although the *recA* gene has a higher taxonomic resolution [16], a 16S rRNA gene based assay is potentially more sensitive because the *B. multivorans* genome contains five copies of the 16S rRNA gene [19]. However, preliminary tests revealed that the 16S rRNA based assay yielded only false positive results, as shown by sequence analysis of the resulting amplicons. A nested PCR design was used to increase the sensitivity of the *recA* PCR assay [20], in which the first PCR was carried out in duplicate and the resulting PCR product was pooled before using it as a template in the second PCR. The in vitro detection limit of this PCR assay was therefore determined to be 5.41 *recA* copies or genome equivalents.

The specificity of the *recA* primers [16] used was previously evaluated [21]. The latter study demonstrated that non-specific primer binding could be eliminated by raising the annealing temperature for the *B. multivorans* assay from 58 °C to 64 °C, resulting in a specificity of 100 % [21]. Additionally, the combination of two different primer pairs in our nested PCR design (specific for the Bcc and *B. multivorans*) further minimized false positive results. Finally, all resulting amplicons were sequenced to ensure that only true positive results were recorded. Whereas this final check yielded satisfactory results for the soil samples (one false positive versus 23 true positives), it revealed a strikingly high number of false positives for the water samples (20 false positives versus 12 true positives). The false positives included samples for which the amplicon could not be sequenced (one soil sample and 15 water samples) or samples for which the resulting sequence did not yield any blast hits (five water samples). These findings demonstrate that this *recA*-based PCR assay in its current form is not suited for routine analysis of water samples, and that one should re-evaluate PCR-based methods in terms of specificity when applying them to environmental samples [22].

To eliminate false negative results caused by PCR inhibitors such as humic acids in the environmental DNA extracts, all samples were screened for the presence of PCR inhibitors using a universal 16S rRNA PCR. In case this universal PCR was inhibited, the DNA extracts were purified using low-melting point agarose plugs. Re-testing the DNA extracts after this purification step demonstrated that this purification procedure was very efficient in removing the humic acids from the DNA extracts, as shown previously by Moreira [23]. Furthermore, optical density should not only be measured at 260 nm because this can lead to an overestimation of the DNA concentration if humic acids are present, but also at 320 nm to quantify these contaminations and to evaluate the quality of environmental DNA extracts properly [24, 25].

Because the few environmental *B. multivorans* isolates that have been reported mainly originated from water samples [12, 14], we hypothesized that water rather than soil was the most likely environmental niche of *B. multivorans*. Our study therefore focused primarily on water samples. However, only 12 out of 112 water samples (11 %) examined were *B. multivorans* PCR positive, in contrast to 23 out of 25 soil samples (92 %) examined. Despite the small number of soil samples, binomial logistic regression showed a highly significant effect (*p* < .001) of the type of sample (Model 1, Table 1) and predicted that there was a 96 times bigger chance to detect *B. multivorans* in soil than in water samples. This result also seemed to suggest that, if present, *B. multivorans* can be isolated more easily from water than from soil samples. Accordingly, Miller et al. [17] showed that although a high percentage of soil samples was PCR positive for Bcc, none of these samples yielded Bcc isolates, demonstrating that cultivation-dependent recovery of Bcc bacteria likely underestimates their prevalence in environmental samples. However, the detection of *B. multivorans* DNA does not necessarily imply that viable target organisms were present at the moment samples were taken [26].

Binomial logistic regression showed that the probability of detecting *B. multivorans* in water samples was higher in streams (CRS) compared to swimming and recreational waters (SRW), and in water samples with a higher pH (Model 2, Table 2). Since pH is known to be an important predictor of bacterial diversity in soil [27, 28], and *Burkholderia* bacteria have been shown to be acid tolerant [29], we expected to detect more *B. multivorans* in acidic versus alkaline waters. The opposite findings of the present study again may suggest that water is not the natural reservoir of *B. multivorans*. However, the number of PCR positive water samples was rather small and therefore one should be careful when extrapolating the results of our binomial logistic regression model 2.

Our finding that *B. multivorans* is widely distributed in soil samples contrasts with the results of previous isolation campaigns, but nevertheless agrees with the notion that the soil environment typically harbors large-genome sized organisms [30, 31]. As *B. multivorans* harbors a ~6.5 Mb genome, it is equipped with the metabolic versatility needed to thrive in a complex, variable environment such as soil. Future research could focus on the genome biology of this organism, and try to infer the lifestyle of this organism based on genome data [32, 33].

## Conclusions

In summary, we applied a *recA*-based PCR assay that demonstrated that *B. multivorans* DNA is widely distributed in soil samples but only occasionally in water samples in Flanders, Belgium. As for all Bcc bacteria it is unclear if and how this mere observation should be implemented in infection control guidelines. Our study also demonstrated that the presently available *B. multivorans recA* primers lack specificity when applied to the analysis of water samples.

## Methods

### Samples

Water (*n* = 112) and soil (*n* = 27) samples were taken from August to October 2013 in Flanders, Belgium (Additional file 1). For the water samples, an autoclaved 1 L Duran bottle was opened and filled 10 cm below the water surface. Per sample, three times 150 ml was filtered using a Nalgene vacuum filter funnel and cellulose nitrate membrane filters with 0.45 μm pore size and 47 mm diameter (Thermo Scientific). For the soil samples, samples were taken 2 cm below the soil surface using a sterile spoon, and collected in sterile falcon tubes. Per sample, 1 g of soil was homogenized in 9 ml phosphate-buffered resuspension buffer (0.15 M NaCl, 10 mM EDTA, 0.1 M phosphate buffer, pH 8.0) using a Stomacher blender for 30 s at 230 rpm. Three times 1 ml of soil suspension was transferred to an Eppendorf tube and centrifuged for 5 min at 13,000 rpm (17,949 g) before removing the supernatant. Filters and soil pellets were stored at −20 °C until DNA extraction.

For each sample, sampling date, address, region (i.e., West-Vlaanderen [WV], Oost-Vlaanderen [OV], Limburg [L], Vlaams-Brabant [VB] or Antwerpen [A]), and class (i.e., swimming or recreational water [SRW], canal-river-stream [CRS] or other [O]) were recorded (Additional file 2). Swimming and recreational waters were those under surveillance of the Flemish Environment Agency (www.kwaliteitzwemwater.be). For water samples, pH and temperature were measured on site. For soil samples, pH was measured after dissolving 10 g of soil in 50 ml distilled water and magnetic stirring for 10 min.

### DNA extraction from environmental samples and quality assessment

Prior to DNA extraction, filters with biological material from water samples were cut into smaller pieces with sterilized scissors. Total DNA was extracted in triplicate from the filters and soil pellets (three per sample) following the protocol for Gram-negative bacteria of Pitcher et al. [34]. DNA pellets were dissolved by adding 50 or 100 μl TE buffer depending on the size of DNA pellet, and left to dissolve overnight at 4 °C. RNA was degraded by adding 2.5 or 5 μl RNase (2 mg/ml) for pellets dissolved in 50 or 100 μl TE, respectively, and incubating at 37 °C for 1 h.

The quality and quantity of the extracted DNA were examined by measuring optical densities (OD) at 234 nm, 260 nm, 280 nm and 320 nm [24, 25] with a SpectraMax Plus 384 spectrophotometer. DNA was considered of acceptable quality if the OD_260/280_ ratio was higher than 1.7, the OD_234/260_ ratio was smaller than 1 and the OD_320/260_ ratio was smaller than 0.15. If both quality and quantity of the three DNA extraction replicates per sample were similar, these replicates were pooled. DNA fragmentation and RNA contamination was assessed by agarose (1 %) gel electrophoresis and EtBr staining.

To test for the presence of PCR inhibitors, DNA extracts were subjected to a 16S rRNA amplification PCR with universal primers ARI C/T (5′-CTG GCT CAG GAY GAA CGC TG-3′) and pH (5′-AAG GAG GTG ATC CAG CCG CA-3′). The PCR mix contained 1× CorelLoad PCR buffer (Qiagen), 0.2 mM dNTP (Applied Biosystems), 0.5 U AmpliTaq (Applied Biosystems) 0.1 μM of both primers and 200 ng/μl BSA (Roche). For each sample, 2 μl DNA was added to 23 μl PCR master mix. *B. multivorans* R-20526 DNA and sterile MQ were used as positive and negative control, respectively. PCR was performed using a MJ Research PTC-100 thermal cycler. Initial denaturation for 5 min at 95 °C was followed by 3 cycles of 1 min at 95 °C, 2 min 15 s at 55 °C and 1 min 15 s at 72 °C, another 30 cycles of 35 s at 95 °C, 1 min 15 s at 55 °C and 1 min 15 s at 72 °C, and a final elongation for 7 min at 72 °C. The presence of amplicons was verified via agarose (1 %) gel electrophoresis with SmartLadder (Eurogentec) as molecular size marker and EtBr staining.

If the OD_320/260_ ratio was higher than 0.15 and/or no universal 16S rRNA amplicon could be obtained, an extra purification step using agarose plugs was performed to remove humic acids and other PCR inhibiting contaminants [23]. After purification using plugs, DNA extracts were diluted 5× in TE buffer. Only DNA extracts for which a universal 16S rRNA amplicon could be obtained, were subjected to the *B. multivorans* PCR assay.

### Preliminary experiment

A preliminary experiment compared the specificity of the *B. multivorans*-specific primers that were based on the *recA* and 16S rRNA gene and were available from previous studies [15, 16]. Sampling of two water samples, DNA extraction and quality assessment of the DNA extracts was performed as described above. For the nested *B. multivorans* 16S rRNA PCR assay, PCR products of the first, universal 16S rRNA PCR were used as template in a second PCR in which *B. multivorans*-specific primers BC-GII (5′- AGG CGG TCT GTT AAG ACA -3′) and BC-R (5′- AGC ACT CCC GAA TCT CTT -3′) were used [15]. The second PCR mix was identical to the first PCR mix, except for a lower BSA concentration (50 ng/μl). For each sample, 2 μl PCR product of the first PCR was added to 23 μl PCR master mix. The positive (*B. multivorans* R-20526) and negative (blank) control of the first PCR (5 μl) were also transferred as template into the second PCR. The thermal cycling program was identical to that of the first PCR. The presence of amplicons was verified via agarose (1 %) gel electrophoresis with SmartLadder (Eurogentec) as molecular size marker and EtBr staining. If an amplicon (445 bp) was visible in the second PCR, it was sequenced using the BC-GII and BC-R primers as described previously [35] to exclude false positive results. The *recA* based *B. multivorans* PCR assay was performed as described below.

### Nested *recA* PCR assay for *B. multivorans*

In a first PCR, Bcc-specific *recA* primers recA-01-F (5′-GAT AGC AAG AAG GGC TCC-3′) and recA-02-R (5′-CTC TTC TTC GTC CAT CGC CTC-3′) were used [36]. The PCR mix contained 1× CorelLoad PCR buffer (Qiagen), 0.25 mM dNTP (Applied Biosystems), 1 U Taq (Qiagen), 0.5 μM of both primers, 1× Q-solution (Qiagen) and 200 ng/μl BSA (Roche). For each sample, 2 μl DNA was added to 23 μl PCR master mix. *B. multivorans* R-20526 DNA and sterile MQ were used as positive and negative control, respectively. This PCR reaction was setup in duplicate for each sample to enable pooling and to increase sensitivity. PCR was performed using a MJ Research PTC-100 thermal cycler. Initial denaturation for 2 min at 94 °C was followed by 35 cycles of 30 s at 94 °C, 45 s at 58 °C and 1 min at 72 °C, and a final elongation for 10 min at 72 °C. PCR product of the duplicate reactions for each sample were pooled using filter tips and used as template in the second PCR, in which *B. multivorans*-specific primers BCRBM1 (5′-CGG CGT CAA CGT GCC GGA T-3′) and BCRBM2 (5′-TCC ATC GCC TCG GCT TCG T-3′) were used [16]. The second PCR mix was identical to the first PCR mix, except for a lower BSA concentration (50 ng/μl) and a higher primer concentration (1 μM). For each sample, 5 μl of pooled PCR product from the first PCR was added to 20 μl PCR master mix. The positive (*B. multivorans* R-20526) and negative (blank) control of the first PCR (5 μl) were also transferred as template into the second PCR. Thermal cycling was identical to the first PCR, except that the annealing temperature was 64 °C instead of 58 °C [21]. The presence of amplicons was verified via agarose (1 %) gel electrophoresis with SmartLadder (Eurogentec) as molecular size marker and EtBr staining. The nested *recA* PCR assay was performed twice for each sample. If the results for the two runs were not the same, the assay was performed a third time. If an amplicon (714 bp) was visible in the second *B. multivorans*-specific PCR, it was sequenced using the BCRBM1 and BCRBM2 primers as described previously [35] to exclude false positive results. Only if at least for one of the replicate runs an amplicon from the second PCR was sequenced that showed at least 97 % similarity to the *recA* sequence of *B. multivorans* ATCC 17616, the sample was considered a true positive for the detection of *B. multivorans*.

To determine the detection limit of this PCR assay, it was applied on serial dilutions of genomic DNA from *B. multivorans* R-20526, a strain for which whole-genome sequencing data is available (BioProject PRJNA234537). The mean weight for an AT and GC base pair is 615.3830 Da and 616.3711 Da, respectively [37]. Given the GC content of this genome of about 68 %, and ignoring the presence of modified nucleotides, the mean relative weight of one base pair of R-20526 is 616.0549 Da or 1.023 × 10^−9^ pg (1 Da = 1.660539 × 10^−24^ g). Given the genome size of 6.5 Mb, one genome of R-20526 contains 6.65 × 10^−6^ ng of DNA, or 1 ng of DNA contains 1.50 × 10^5^ genome equivalents. The undiluted genomic DNA stock of strain R-20526 contained 225 ng/μl DNA, as measured with the Promega QuantiFluor ONE dsDNA system, or 3.38 × 10^7^ genomic DNA equivalents per μl. The PCR assay was first applied on a 50× dilution series (in TE buffer) to find the approximate fading range. The highest 50× dilution for which an amplicon could be obtained was then used to make a twofold dilution series to find the limit of detection. The PCR assay was performed three times for each dilution and the detection limit was defined as the highest twofold dilution that tested positive in all three runs. Considering the *recA* gene is a single-copy gene for *B. multivorans* R-20526, the detection limit was calculated from the measured DNA concentration and calculated DNA content.

### Statistical data analysis

Statistical data analysis was performed using R version 3.1.2 in RStudio (version 0.98.1091), with the following packages: car, ggplot2, and MASS. Binomial logistic regression was used to test which variables (type, region, class, pH, temperature) were significant predictor variables for the outcome variable, i.e., *B. multivorans* detection (Bm). Type, class, region, and Bm were coded as factors, and the level with the most cases was chosen as reference category. Temperature and pH were coded as numeric variables, and centered around the mean to reduce standard error (SE). Backward stepwise model selection was applied to select the best fitting models.

## Abbreviations

*Bcc Burkholderia cepacia* complex, *CF* cystic fibrosis

## Additional files

Additional file 1: sampling map. Map showing all sampling locations as produced by the ggmap and ggplot2 packages in R (own figure). Bm, *B. multivorans*. (pdf). (PDF 913 kb) Additional file 2: sample data. Bcc, *Burkholderia cepacia* complex PCR positive; Bm, *B. multivorans* PCR positive; NA, not available. WV, West-Vlaanderen; OV, Oost-Vlaanderen; L, Limburg; VB, Vlaams-Brabant; A, Antwerpen. SRW, swimming or recreational water; CRS, canal-river-stream; O, other. (xlsx). (XLSX 17 kb)
