# Supplementary figures and images for: PCR detection of Burkholderia multivorans in water and soil samples
Source: BMC Microbiol. 2016 Aug 12;16:184. doi: 10.1186/s12866-016-0801-9 (PMC4981952; doi:10.1186/s12866-016-0801-9)

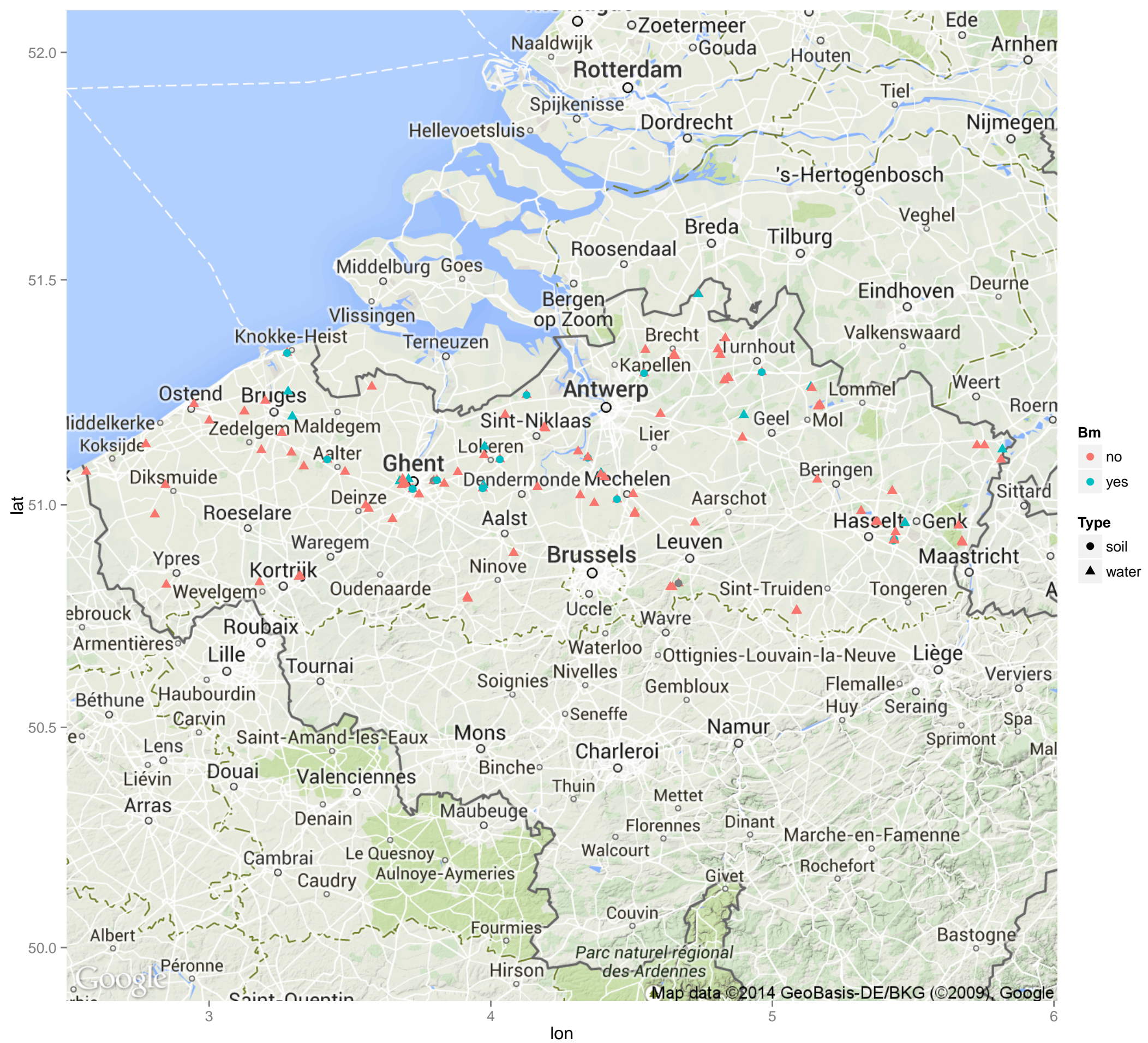

Supplement: Additional file 1: sampling map. — Map showing all sampling locations as produced by the ggmap and ggplot2 packages in R (own figure). Bm, B. multivorans. (pdf). (PDF 913 kb) [file 12866_2016_801_MOESM1_ESM.pdf]
